# Nitrate Promotes Capsaicin Accumulation in *Capsicum chinense* Immobilized Placentas

**DOI:** 10.1155/2015/794084

**Published:** 2015-02-01

**Authors:** Jeanny G. Aldana-Iuit, Enrique Sauri-Duch, María de Lourdes Miranda-Ham, Lizbeth A. Castro-Concha, Luis F. Cuevas-Glory, Felipe A. Vázquez-Flota

**Affiliations:** ^1^Instituto Tecnológico de Mérida, Km. 5 Carretera Mérida-Progreso, 97118 Mérida, YUC, Mexico; ^2^Unidad de Bioquímica y Biología Molecular de Plantas, Centro de Investigación Científica de Yucatán, Calle 43 No. 130, Chuburná de Hidalgo, 97205 Mérida, YUC, Mexico

## Abstract

In chili pepper's pods, placental tissue is responsible for the synthesis of capsaicinoids (CAPs), the compounds behind their typical hot flavor or pungency, which are synthesized from phenylalanine and branched amino acids. Placental tissue sections from Habanero peppers (*Capsicum chinense* Jacq.) were immobilized in a calcium alginate matrix and cultured *in vitro*, either continuously for 28 days or during two 14-day subculture periods. Immobilized placental tissue remained viable and metabolically active for up to 21 days, indicating its ability to interact with media components. CAPs contents abruptly decreased during the first 7 days in culture, probably due to structural damage to the placenta as revealed by scanning electron microcopy. CAPs levels remained low throughout the entire culture period, even though a slight recovery was noted in subcultured placentas. However, doubling the medium's nitrate content (from 40 to 80 mM) resulted in an important increment, reaching values similar to those of intact pod's placentas. These data suggest that isolated pepper placentas cultured *in vitro* remain metabolically active and are capable of metabolizing inorganic nitrogen sources, first into amino acids and, then, channeling them to CAP synthesis.

## 1. Introduction

**Figure 1:**
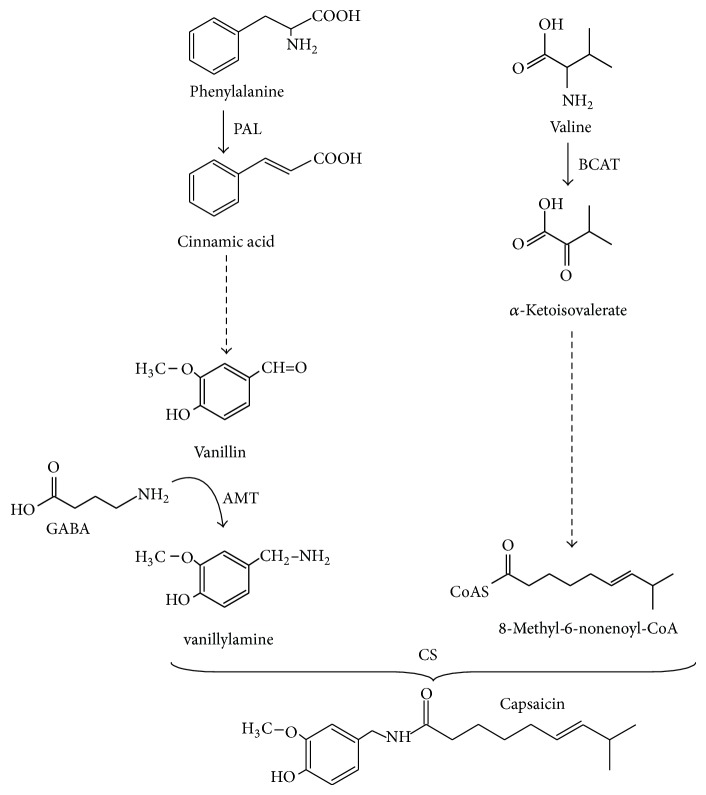
Involvement of nitrogen or nitrogenous metabolites in capsaicin biosynthesis. AMT, vanillin aminotransferase; BCAT, branched amino acid aminotransferase; PAL, phenylalanine ammonia-lyase.

In plants, the placenta is the filmy tissue that holds the seeds inside the fruit. In the* Capsicum* genus, it also produces and accumulates capsaicinoids (CAPs), compounds responsible for the burning flavor of peppers [1]. CAPs are formed through the convergence of two biosynthetic pathways, one from phenylalanine and the other from branched amino acids, either valine or leucine [2–5]. Due to the interest in CAPs biosynthesis,* in vitro* cell cultures from different* Capsicum* species have been employed as a model to study the basis for its regulation; however, nondifferentiated cell suspensions accumulate very low CAP amounts compared to intact placental tissues [6, 7], which is maybe due to the lack of the required specialized structures. In intact placentas, CAPs are stored in vesicles derived from the epithelial cells [1]. Since CAP biosynthesis is exclusively located in the placental tissue, the use of sections of this tissue cultured* in vitro* has been envisioned as an alternative system [7, 8]. Two methods for the* in vitro* culture of pepper placentas have been used: free and immobilized tissues. Free placentas cannot maintain their integrity as they become swollen and disintegrate after short periods in culture. This effect can be avoided by immobilizing the tissue [9]. Tissue immobilization consists in entrapping sections within an inert support, keeping it alive and functional in a medium with mineral salts and organic compounds under sterile conditions. Immobilized tissues will remain metabolically active, although growth and development have been arrested. This technique has been widely employed for the production of plant fine chemicals, as well as for their functional preservation [9]. On the other hand, mineral nutrition can affect CAPs accumulation and, thence, pungency in chili peppers. Moreover, these effects can be exerted at various levels, such as modifying the biosynthetic ability of the participating tissues or promoting their biomass accumulation [10, 11]. Three nitrogenous molecules are involved in CAPs synthesis (Figure 1). These are: Phenylalanine, which provides the phenolic moiety, a branched amino acid, either valine or leucine, that contributes with the lateral aliphatic chain, and *γ*-aminobutyric acid (GABA), the amino donor required to form vanillylamine from vanillin ([12]; Figure 1). Hence, as expected, CAP contents in pepper pods respond to external nitrogen availability. In fact, a positive correlation between nitrate and CAPs contents occurs in placentas from Habanero pepper pods [11]. To further assess the dependence of CAP biosynthesis on nitrogen availability, isolated Habanero pepper placentas were cultured* in vitro* and exposed to different nitrate doses. Previous to nitrogen treatments, placentas were immobilized in calcium alginate, in order to keep tissue integrity, and metabolic functionality was evaluated.

## 2. Materials and Methods

### 2.1. Biological Materials

Placentas were obtained from pods of an orange accession of Habanero pepper (*Capsicum chinense* Jacq.) collected from commercial plantations in Yucatán, México [13, 14]. Cell suspensions of* C. chinense* were maintained at the same conditions described previously [15].

### 2.2. Tissue Immobilization Process

Immature pods (16–20 days after anthesis,* ca. *3 × 2 cm; long × wide) were used shortly after harvest. Pods were disinfested with ethanol (80%) and commercial bleach (50% equivalent to 3 mg/L of sodium hypochlorite), before placentas were excised with scalpel and tweezers. Placentas were kept in sterile water prior to immobilization or transfer to the culture medium. Sections of placental tissue (0.25 cm^2^) were suspended in a beaker containing 2.5% sodium alginate (Sigma) at room temperature inside a flow cabinet with constant and gentle stirring in order to distribute them homogenously in the vessel. To obtain the alginate beads, a blunt-tip 10 mL glass pipette was used to drop* ca.* 1 mL of this suspension into cold 1% CaCl_2_ under constant, gentle stirring. Immobilized placentas were allowed to stand for 90 minutes at 4°C, washed with cold, distilled water to eliminate excess calcium, and transferred to 250 mL Erlenmeyer flasks containing 40 mL of Murashige and Skoog (MS) medium without growth regulators. Each flask received* ca*. 40 alginate beads with tissue sections entrapped and were kept for a 28-day culture period, either continuously or with a renewal of medium after the first 14 days.

### 2.3. Nitrate Treatments

Nitrate in MS medium is supplied as a mixture of NH_4_NO_3_ and KNO_3_, accounting for a total concentration of 40 mM. In order to promote CAPs accumulation, immobilized placentas were exposed to different nitrate doses: 20, 40 (normal content), 60, or 80 mM. Potassium nitrate was used in the 20 and 40 mM treatments, so no potassium compensation was required in these treatments. Nevertheless, ammonium content was compensated as NH_4_Cl when total nitrate was reduced to 20 mM. To avoid an excess of potassium ions, extra nitrate in 60 and 80 mM treatments was supplied as NaNO_3_. Any side effects resulting from extra chloride and sodium ions were analyzed by exposing the immobilized placentas to 20 mM NaCl added to standard MS medium. After 14 days, immobilized placenta sections were either subcultured to fresh media with the same nitrate doses or kept in the original media for an extra 14 days. In both cases, total culture period was 28 days. Similar experiments were performed with cell suspensions for comparative analysis. Two-week old cell cultures were transferred to fresh MS medium with 20, 40 (control), 60, or 80 mM nitrate, adjusting to one gram of cells (FW) per 40 mL of media contained in 250 mL Erlenmeyer flasks.

### 2.4. Analytical Procedures

After treatments, immobilized placentas were collected, frozen in liquid nitrogen, and kept at −80°C until analysis. To estimate tissue viability throughout the culture period, total soluble sugar and amino acids were quantified in aqueous extracts following standard procedures [8]. Phenylalanine ammonia-lyase (PAL) activity that catalyzes the first reaction leading to CAP biosynthesis was analyzed [15] to evaluate possible effects on this pathway. Protein extracts were obtained as follows: beads were ground to a fine powder and mixed with appropriate extraction buffers [8, 15]. The slurry was first filtered through four layers of cheesecloth and then centrifuged to eliminate alginate residues. CAPs were evaluated in methanolic extracts after chromatographic separation as described previously [16].

### 2.5. Scanning Electron Microscopy (SEM)

Immobilized placental tissue was excised from pods with scalpel and tweezers and fixed with FAA (10% formaldehyde, 50% ethanol, and 5% acetic acid) as described elsewhere [17]. Afterwards, tissues were dehydrated with an increasing ethanol series (from 30 to 100%), and solvent-exchanged with liquid CO_2_, and dried to critical-point (Penten Vacuum Desk). Dehydrated placental tissues were then mounted on aluminum stubs and twice coated with gold emulsion (Sputter-coater: Sandri-795). Section surface was examined using a JEOL 6360LV scanning electron microscope (SEM).

### 2.6. Statistical Analysis

Data were analyzed using one- or two-way ANOVA and means were compared using Tukey's HSD test at *P* < 0.05, with the Statsoft Statistica 7 package. Results shown correspond to triplicates taken from one single experiment since trends were similar in two independent experiments. Mean values are presented with their standard deviation.

## 3. Results and Discussion

Immobilized placentas were cultured* in vitro* for either a single, continuous 28-day period (nonsubcultured) or through two 14-day culture periods (one subculture).

**Figure 2:**
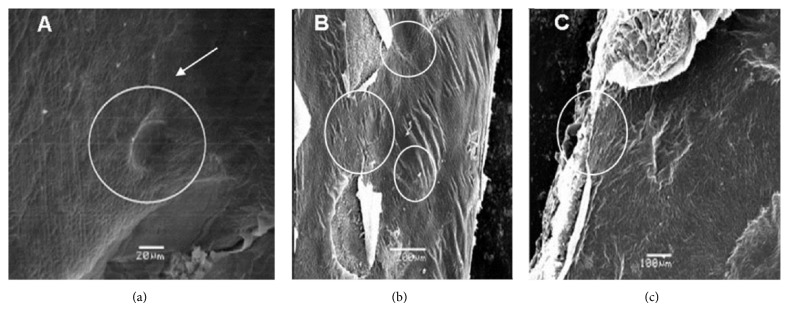
Scanning electron micrographs of immobilized placentas after 7 (a), 14 (b), and 21 (c) days in* in vitro* culture. Circle-enclosed areas with the presence of blisters, either intact (a) or damaged (b and c).

**Figure 3:**
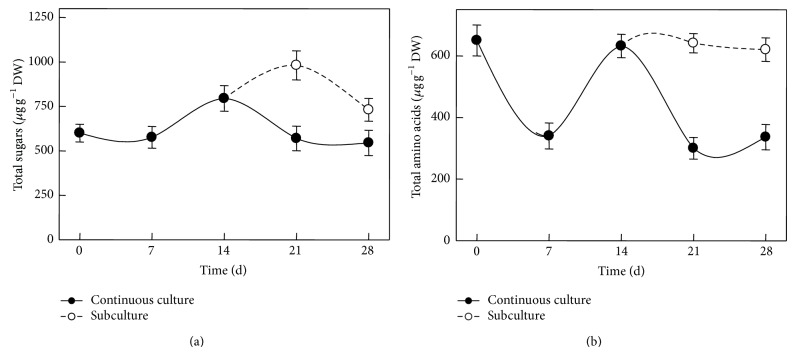
Total sugars (a) and amino acids (b) contents in immobilized placentas cultured* in vitro* for continuous 28 days (continuous lines) or with medium renewal after 14 days (dashed lines; subculture). Means of 5 replicates ± SD.

In intact placentas, CAPs are accumulated in vesicles derived from swollen epithelial cells (Figure 2(a); [1]). The structure of such vesicles was observed by electron microscopy (Figure 2). Although most of these vesicles were preserved in encapsulated placentas, manipulation may have caused some physical damage (Figure 2(b)). No further damage to these structures was recorded throughout the culture period, neither the formation of new ones (Figures 2(b) and 2(c)). Nevertheless, CAPs recovered from the medium were in very low concentrations, perhaps due to their oxidation, as has been previously proposed [18]. This fact may explain the cause of lower CAPs contents in immobilized placentas when compared to the intact tissue excised from pepper pods (*ca*. 0.5 versus 6.6 mg g^−1^ DW). In order to determine if immobilized placentas were still metabolically active, soluble sugars and amino acids contents were analyzed in both, those maintained in a 28-day continuous culture or with a 14-day subculture (Figure 3). During the first seven days of culture, sugars remained unchanged compared to the initial contents, increasing shortly after. However, further increase was only noticed in subcultured placentas (Figure 3(a)). Soluble amino acids sharply decreased in the first seven days and then recovered their initial values. In the case of those placentas that were not subcultured, the contents declined again sharply (Figure 3(b)).

**Figure 4:**
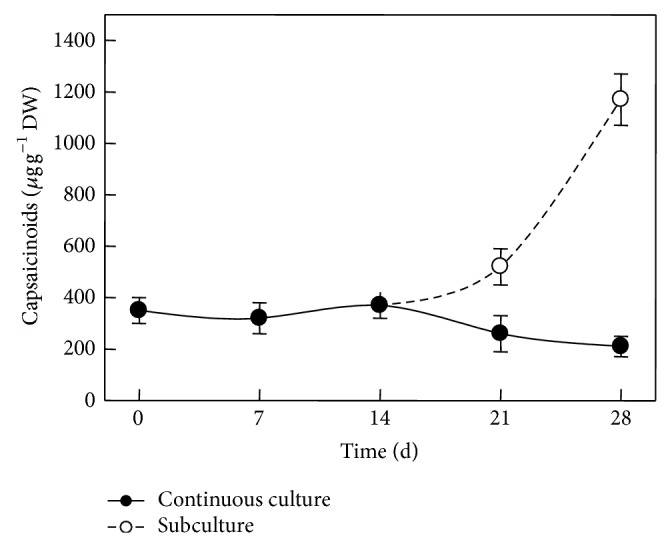
Capsaicinoid content in immobilized placentas cultured* in vitro* for 28 days continuous lines) or with medium renewal after 14 days (dashed lines; subculture). Means of 5 replicates ± SD.

**Figure 5:**
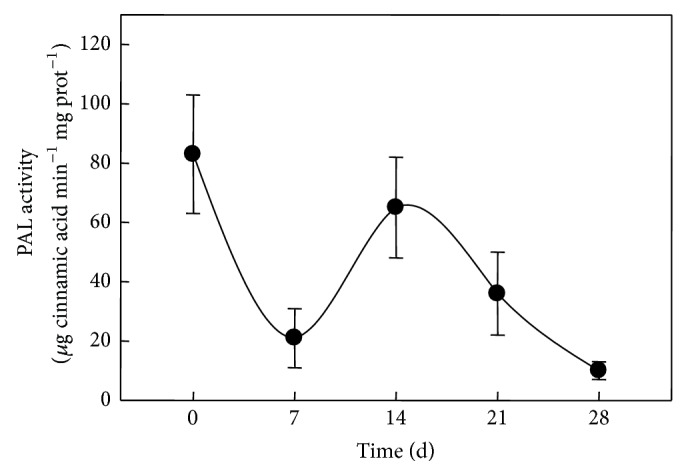
Phenylalanine ammonia-lyase specific activity in intact placentas and after 7, 14, 21, and 28 days of* in vitro* culture. Means of 5 replicates ± SD.

In this way, renewal of the medium might have stopped the decline in these metabolites levels, suggesting that the encapsulated tissues under* in vitro* conditions were able to interact with the surrounding medium. Moreover, nondetached placentas from pods stored under similar conditions to those in* in vitro* cultures (culture room conditions) did not maintain the initial values through similar time periods (data not shown), implying that decay in metabolic activity was occurring in those tissues that were not receiving an external supply of nutrients. Interestingly, CAPs accumulation also increased in response to medium renewal (Figure 4). Phenylalanine ammonia-lyase (PAL) activity, the first enzyme of the phenylpropanoid pathway and thus initiating the formation of the phenolic moiety of CAPs, decreased to a fourth of that found in intact placentas during the first seven days in culture (Figure 5). However, PAL activity levels reverted to their initial values in intact tissues after 14 days, although such levels were not maintained afterwards (Figure 5). The modulation pattern shown by PAL suggests that immobilized placentas might maintain the ability to produce the phenolic compounds required for CAPs biosynthesis, although no direct correlation between PAL activity (Figure 5) and CAPs accumulation (Figure 4) was observed. This result contrasts with what was found for suspension cells from* C. annuum*, where an increase in PAL activity correlated to an important accumulation of both, CAPs and phenolic intermediaries [7], suggesting that regulatory controls for CAPs synthesis in intact placentas may be exerted at different points than in cell cultures [19].

### 3.1. Nitrate Increases CAPs Accumulation in Immobilized Placentas of Habanero Pepper

**Figure 6:**
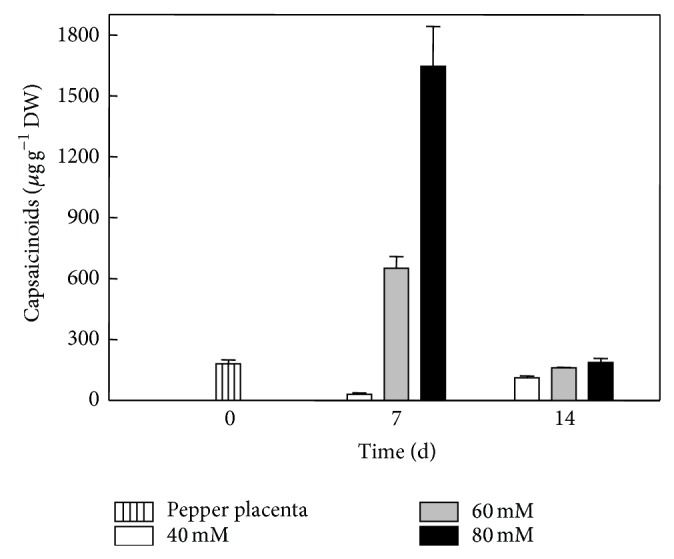
Capsaicinoid content in placentas cultured* in vitro* under three nitrogen regimes for 7 or 14 days. White bars, 40 mM nitrate; gray bars, 60 mM nitrate; and black bars, 80 mM nitrate. Means of 5 replicates ± SD. Dashed bar at 0 days represents initial capsaicinoid contents.

**Figure 7:**
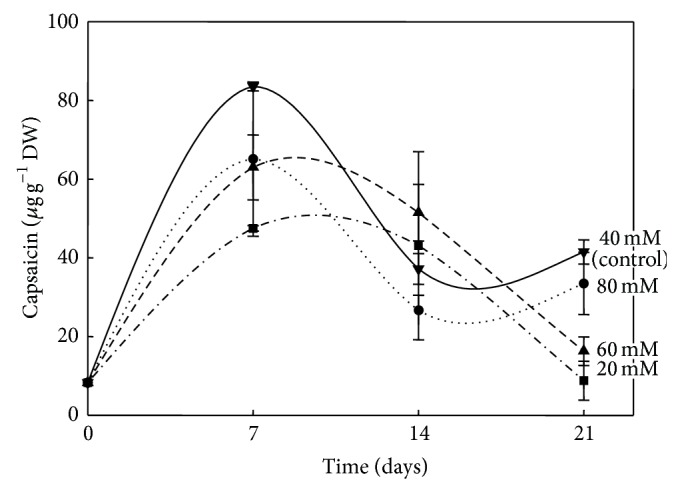
Capsaicin content in* C. chinense* cell cultures exposed to different nitrate doses. Two-week cultures were transferred to fresh medium with 20, 40 (control), 60, or 80 mM nitrate. Means of 5 replicates ± SD.

Immobilized pepper placentas were able to remain metabolically active and to produce CAPs throughout a 21-day culture period (Figures 3 and 4). When nitrogen content in the media was modified, cultured placentas showed concomitant variations in CAPs contents. After seven days in culture, placentas exposed to half the normal dose of MS nitrate (20 mM) accumulated undetectable amounts of CAPs (data not shown). In contrast, when the full MS nitrate content (40 mM) was employed, placentas accumulated up to 0.3 mg CAP g^−1^ DW. A sharp increase in CAPs level was detected when available nitrate increased to 60 mM (6.4 mg g^−1^ DW) or 80 mM (15.6 mg g^−1^ DW) for seven days (Figure 6). Longer exposures to these nitrate levels were detrimental to CAPs accumulation since there were decreases of over 80–85% in the levels observed before. These results confirm that immobilized placentas cultured* in vitro* were able to synthesize CAPs and suggest that externally supplied nitrate may be converted into the required amino acids for this process. Activation of primary nitrogen assimilation in response to modification of the nitrogen source has been demonstrated in placental tissues cultured* in vitro* [8]. It is noteworthy to point out that the increase in CAPs levels when nitrate concentration was increased from 60 to 80 mM was lower than that observed between 40 and 60 mM of nitrate. Interestingly, undifferentiated* C. chinense* cell cultures did not respond in a similar fashion to variations in the nitrogen source (Figure 7). This may imply that other factors than nitrogen availability, such as the presence of organized tissue, could limit CAPs accumulation in* in vitro* conditions.

## 4. Concluding Remarks

Immobilized placentas of Habanero pepper could be kept viable and metabolically active for up to 21 days under the assayed conditions. Interestingly, as it has been recorded for intact placentas in whole pepper pods [11],* in vitro* cultured placentas increased their CAP contents when nitrate availability increased (Figure 6). These observations suggest that isolated placentas can interact with the components of the medium and that inorganic nitrogen can be incorporated, first into amino acids and, then, to CAPs. In this sense, an active primary assimilation of nitrogen into amino acids has been determined in placentas of Habanero peppers [8]. Therefore, given the proper conditions, placental tissues are able to autonomously function to maintain complex metabolic pathways, such as those involved in secondary metabolism.
